# Botulinum Toxin: From Poison to Possible Treatment for Spasticity in Spinal Cord Injury

**DOI:** 10.3390/ijms22094886

**Published:** 2021-05-05

**Authors:** Ramiro Palazón-García, Ana María Benavente-Valdepeñas

**Affiliations:** 1 Physical Medicine and Rehabilitation Department, Hospital Nacional de Parapléjicos, 45004 Toledo, Spain; 2 Physical Medicine and Rehabilitation Department, Hospital Virgen de la Salud, 45004 Toledo, Spain; anabenaval@yahoo.es

**Keywords:** botulism, botulinum neurotoxin, spasticity, spinal cord injury

## Abstract

Botulism has been known for about three centuries, and since its discovery, botulinum toxin has been considered one of the most powerful toxins. However, throughout the 20th century, several medical applications have been discovered, among which the treatment of spasticity stands out. Botulinum toxin is the only pharmacological treatment recommended for spasticity of strokes and cerebral palsy. Although its use as an adjuvant treatment against spasticity in spinal cord injuries is not even approved, botulinum toxin is being used against such injuries. This article describes the advances that have been made throughout history leading to the therapeutic use of botulinum toxin and, in particular, its application to the treatment of spasticity in spinal cord injury.

Botulinum toxin serotype A (BoNT/A) is produced by the Clostridium botulinum bacterium. It is a metalloprotease which, in nerve endings, proteolytically cleaves synaptosomal associated protein-25 (SNAP-25) to inhibit the fusion of the synaptic vesicle with the presynaptic membrane of the axon terminal, and thus ultimately relax the muscle [1]. Botulinum neurotoxins (BoNTs) represent the most poisonous biological substances known today because they produce severe neurological diseases, such as botulism [2].

Although botulinum toxin was initially described as a potent poison, its therapeutic use for spasticity, pain, and other disorders has become widespread in the last 40 years [3]. Our objective is to show the evolution that has occurred since botulinum toxin was considered a dangerous poison until it can now be used to treat spasticity in spinal cord injuries (SCI) and show how this treatment can be performed.

## 1. From Poison to Remedy

Botulism presents a clinical picture characterized by symmetrical cranial nerve palsies followed by descending, symmetric flaccid paralysis of voluntary muscles, which may progress to severe respiratory failure and death [4]. There are four main forms: food-borne, wound, infant botulism, and adult intestinal toxemia. All are related to the entry into the organism of toxins of C. botulinum and less frequently of C. barati, C. butyrricum, C. argentinense, and C. sporogenes. The route of entry and spread is usually enteral, although the toxicity is neurological [2,5,6].

Outbreaks of botulism have been described throughout history. In general, they have been related to poorly preserved food (sausages, ham, fish) or home-canned food [7]. Although botulism had probably previously existed and had been confused with other diseases, it is estimated that the first case described is from the year 1735. At the end of the 18th century in Southwestern Germany, the consumption of blood sausages began to be associated with the appearance of gastrointestinal problems, diplopia, and mydriasis, and this was called “sausage poisoning”, suggesting that it could be prevented by boiling them properly [8].

The first reports were published in 1815 by a health officer named J. Steinbuch and by a medical officer named Justinus Kerner, who was the one who made the most detailed description from 1817 of the then called sausage poison. Kerner had been studying the “fatty acid” or sausage poison through analysis of patients with botulism, autopsies of the deceased, and experiments by inoculating extracts of infected tissues into animals. Although he could not isolate the toxin, he could describe that the way it acted was by an interruption of the peripheral and autonomic nervous signal transmission, and because of this work, botulism was known at this time as Kerner’s disease. Despite considering it a dangerous poison, he had already postulated that it could be used as a medicine against phenomena related to overactivity (sweating crisis, chorea) [7].

In December 1895, there was a terrible outbreak of botulism in a small Belgian town called Ellezelles because at a lunch after a funeral, 34 people got together to eat pickled and smoked ham. All of them were infected, coursing with progressive paralysis, 3 of them dying and 10 nearly dying. A microbiologist named Emile Van Ermengem analyzed the food and patients and was able to isolate an anaerobic microorganism that he called Bacillus botulinum [9].

Another microbiologist named Georgina Burke had access to the strains involved in the poisoning described by Van Ermengem and to other strains described in other intoxication and found that they had different serologic characteristics. Those of Van Ermengem were non-proteolytic and she classified them as type B, and the others were proteolytic and she classified them as A [10,11]. The name “B. botulinum” was changed to Clostridium botulinum when the aerobic Bacillus genus was separated from the anaerobic Clostridium genus [12]. After these experiences, data have continued to be collected on botulism outbreaks that have occurred around the world, showing that the most serious and frequent are produced by BoNT/A [13].

After World War II, the mechanism of action of botulinum toxins was described as follows. When botulinum toxin lost its medullary sheath on entry into the endplate, it was irreversibly fixed to the fine nerve fibers. Consequently, no release of acetylcholine took place, and then the transmission of the impulse through those fibers was abolished, resulting in the observed neuro-muscular block. It was also found that a much higher dose of toxin B than toxin A was required to achieve the same paralyzing effect [14].

The first therapeutic use of botulinum toxin was reported by ophthalmologist Alan Scott to treat strabismus in 1973 [15]. Later, it began to be used in dystonia, such as hemifacial spasm or torticollis. In 1989, the FDA approved the indication of BoNT/A for the treatment of blepharospasm, and in the 1990s, the indication for cerebral spasticity (stroke and cerebral palsy) was approved too. Its use in cosmetics and other indications, such as hiperhidrosis, is already recognized. For this reason, since the late 1980s, the main research has focused on the mechanisms of action of botulinum toxins for therapeutic use and the development of new safer and more potent types and formulations of toxins and new therapeutic indications [16]. The first time the botulinum toxin was used in spasticity was in 1989 (Das and Park), and the first randomized, placebo-controlled double-blind studies were published in 1995 [17].

Therefore, throughout the 20th century, our view of botulinum toxin has changed from being a dangerous poison to being a possible remedy for various diseases. One of the examples of the therapeutic utility of the toxin is the treatment of spasticity in spinal cord injury.

## 2. Botulinum Toxin

### 2.1. Botulinum Toxin Structure

BoNT is made up of 2 chains, a heavy one of 100 kDa (H) and a 50 kDa light chain (L) with zinc protease properties. The chains are linked by a disulfide bridge necessary for the toxin to be biologically active [18]. The H chain is made up of two 50 kDa domains: the amino-terminal part, responsible for the translocation of the L chain across the membrane of the endocytic vesicle, and the carboxy-terminal part is for its binding to a polysialoganglioside and the luminal domain of a synaptic vesicle protein in nerve endings [19]. The L chain is a metalloprotease responsible for proteolytic activity with specificity on the three soluble NSF (N-ethylmaleimide-sensitive factor) attachment-protein receptor (SNARE) proteins that are vesicle-associated membrane proteins (VAMP)/synaptobrevin, SNAP-25, and syntaxin [20]. BoNT is genetically encoded together with other proteins, such as nontoxic nonhemagglutinin protein (NTNHA) and a protein related to hemagglutination activity (HA) that may serve to shield it, and together, they make up the so-called progenitor toxin complexes (PTC) [16].

### 2.2. Serotypes

There are seven different types of BoNT that can be distinguished according to different antisera performed with in vitro analysis, and they are designated as A, B, C, D, E, F, and G [21]. Recently nine serotypes have been recognized by adding toxinotypes H and X [5]. Other new types of BoNT have been discovered by new genetic sequencing techniques and have been grouped as subtypes within the classic serotypes with which they share the structure and only differ in some amino acid sequences, naming them with the letter of the serotype and a number (e.g., for serotype BoNT/A there are subtypes including BoNT/A1, BoNT/A2, etc.) [5,16]. Depending on the BoNT serotype, the L chain will exert its proteolytic capacity on different components of the SNARE complex, with specific binding sites for each serotype; BoNT/A specifically cleaves component SNAP-25 [18].

### 2.3. Mechanism of Action

When PTC enter the body, they can be broken down in slightly basic pH media, such as the intestine or in intramuscular fluids, and BoNT is then released [22]. There are five steps described: binding with high affinity to the presynaptic plasma membrane of skeletal and autonomic cholinergic nerve terminals, internalization within an endocytic compartment, translocation of the L chain through the vesicle membrane, breaking of the interchain disulfide bond and release of the L chain in the cytosol, and finally, blockade of acetylcholine release by cleavage of SNAREs [16].

### 2.4. Duration of the Effect

Neuroparalysis in humans occurs within 36–72 h after intramuscular injection, although it can be delayed for 2 weeks, and the peak of greatest intensity is reached at 2–4 weeks [23]. The duration of the effect depends on how long it takes the SNARE complex to become operative again, which can vary depending on [16,24]: the type of BoNT (BoNT/A is the longest), dose (the higher the dose, the longer the duration), mode of administration and type of nerve terminal. Neuromuscular transmission is usually restored within 3–4 months by two independent mechanisms described in in vivo studies in laboratory animals: collateral sprouting, defined as the formation of new synaptic connections with the intact adjacent nerve cells, that begin to appear after 3–4 days and during this process are the only ones capable of releasing acetylcholine, and regenerative sprouting, i.e., the restoration of the anatomical and functional integrity of the nerve terminals that were originally affected by the toxin [23].

## 3. Therapeutic Use for Botulinum Neurotoxins

### 3.1. Current Botulinum Neurotoxin Formulations

Currently, there are clinical trials to investigate whether BoNT/F or BoNT/A2 could be used as therapy in humans. BoNT/A2 has been proven to be more effective than BoNT/A1 at 30 days of injection and less likely to have unintended effects at a distance [25]. However, the presentations marketed now are almost all based on BoNT/A1 and one on BoNT/B1.

There are some BoNT/A-based products that are only marketed in China or Korea, but the main presentations are available worldwide. OnabotulinumtoxinA (ONA) and AbobotulinumtoxinA (ABO) are purified PTC containing BoNT/A1 (pharmacologically active ingredient), NTHTA, and the HA proteins. ONA is a 900 kDa complex marketed as a vacuum-dried powder for reconstitution; it remains usable at 2–8 °C for 36 months. ABO is an 800 kDa complex marketed as a freeze-dried powder for reconstitution, and it remains usable at 2–8 °C for 24 months. IncobotulinumA (INC) contains only the purified BoNT/A1, is a 150 kDa molecule marketed as a freeze-dried powder for reconstitution, and remains usable at room temperature for 36 months. RimabotulinumtoxinB is the only presentation of BoNT/B1, is a 320 kDa molecule, is marketed as a ready-to-use solution, and remains usable at 2–8 °C for 24 months.

The potency of each BoNT formulation is expressed in arbitrary units, with one unit corresponding to 1 LD50 in the mouse bioassay (amount of toxin affecting 50% of animals injected by, i.e., route at defined time post-injection).and; for this reason, the units of each BoNT are not comparable with each other nor can the doses of each BoNT be interchangeable [26]. Despite the possible clinical and economic repercussions, there are very few studies that compare BoNT presentations in terms of which one may be the most appropriate, effective, and affordable according to each pathology. There is a study that suggests that the treatment for cervical dystonia with ABO may be less costly and lead to improved clinical outcomes when compared with ONA. There is another study that suggests that ABO may have more intense and lasting effects, but in all cases, the results are relativized because the calculations are made on the doses recommended by the FDA for each pathology and according to each formulation, which are, therefore, not comparable [27,28].

### 3.2. Clinical Applications

Dystonia.BoNTs can significantly temporarily relieve sustained contractures and repetitive twisting movements, constituting the first-choice treatment for most dystonia [29]. BoNT/A treatment of blepharospasm was approved by the FDA in 1989. Cervical dystonia is the only official indication for BoNT/B.

Spasticity. It is defined as a “disordered sensorimotor control, resulting from an upper motor neuron lesion, presenting as intermittent or sustained involuntary activation of muscles” [30]. It is the most important indication of central nervous system (CNS) disorders. Its efficacy was demonstrated in the early 1990s, although traditionally evidence had only been found that it is effective when used in patients with stroke or infantile cerebral palsy (CP), or for the treatment of hip adductors in patients with multiple sclerosis, and for this reason, these are the only diseases causing spasticity which have treatments approved by the FDA [31,32]. Furthermore, in almost all clinical guidelines for the treatment of CP or post-stroke spasticity, oral antispastic medication is not allowed, and, therefore, the only drug we can use is BoNT. Spasticity caused by other disorders can be treated with BoNTs, although it does not have an official indication, but, in general, it is recommended only if the distribution is focal [33,34]. The discovery of toxin’s retrograde axonal transport to CNS might suggest additional action sites, which in this case would be central (apart from directly injected muscle fibers) but is not yet well studied [35].

Other disorders of the CNS. Neurogenic detrusor overactivity, usually due to spinal cord injury (SCI), is a treatment indication approved by the FDA since 2011 and supported by clinical trials [36]. BoNT can also be used to relax trismus or bruxism secondary to brain damage [37].

Autonomic disorders. BoNT/A has an approved indication for the treatment of severe and persistent primary hyperhidrosis of the axilla [38].

Other urologic disorders. BoNT can be applied in painful bladder or cystitis [39] and for the treatment of urethral sphincter alterations, sphincter pseudodysynergia, or obstructive syndromes [40].

Peripheral facial paralysis. BoNT/A can be used to regain facial symmetry both at rest and during voluntary movement and as a treatment for synkinesis [41].

Pain. The only approved indication for BoNT is chronic migraine, where it acts by interfering with peripheral and possibly central sensitization, and its main role is the block of vasoactive peptides release from trigemino-vascular endings [42]. BoNT has been used successfully in neuropathic pain, mainly of peripheral origin (postherpetic neuralgia, diabetic neuropathy) [43]. It can also be used to treat myofascial pain, temporomandibular disorders, low back pain, Arnold occipital neuralgia and tension neck pain, and idiopathic or dental bruxism [44].

Aesthetics. Cosmetic use has become the most popular application, and there are specific presentations of each formulation for this indication.

### 3.3. Adverse Effects and Other Considerations

The overall rate of possible adverse effects is minimal; effects related to the injection itself (infection, bruising, bleeding) are rare when the injector is skilled and experienced.

The possibility of local diffusion or even leakage into the systemic circulation with distal effects has been described. Diffusion between adjacent muscles wrapped in the same aponeurotic casing (usually synergistic function or similar movement) or between small muscles, such as those of the face, is relatively likely, but distant migration via axonal or hematogenous transport is exceedingly rare [45]. The likelihood of diffusion appears to increase with a higher total dose per muscle. If we are treating spasticity and the diffusion occurs to a synergistic muscle to the target, we could achieve a synergistic effect, but if the diffusion occurs to a different muscle, it would be a real adverse effect because we could weaken a muscle that had no problems previously [46].

Muscle weakness or loss of function may be found due to paralysis of the target organ we are injecting [47].

The possibility of allergic or hypersensitivity reactions has been described but are extremely rare.

Immunogenicity. The production of antitoxin antibodies is postulated, which, rather than detecting them, is suspected when there is a loss of response to the BoNT in prolonged treatments. It is recommended not to exceed the intervals of 3 months for ABO and ONA or 6 weeks in the case of INC to avoid this. The probability of generating antibodies is extremely low, but it is higher with BoNT/B than with BoNT/A. Curiously, the indication for BoNT/B is the loss of efficacy of BoNT/A due to the formation of antibodies [16,48].

The repeated injection of BoNT can maintain and even increase the beneficial effects over time [49], for which a chronic treatment, typical in dystonia, migraine, and spasticity, could be justified. However, there is a potential danger of local effects after repeated injections, such as those seen in the treatment of overactive bladder, where patients no longer responding to BoNT treatment displayed a significant increase in the afferent terminals, likely excitatory, and signs of chronic neurogenic inflammation in the mucosa [50].

Interactions. If the toxin is administered at the same time as anticholinergics or neuromuscular blocking agents, there may be a multiplier effect, such as an overdose, and if it is administered together with aminoglycosides, there is a competition for the cleavage of SNAP-25 that can lead to the loss of effect of any of them [46].

Contraindications. BoNT should not be injected over a local infection or in case of systemic infection. Injection is not recommended in the case of active bleeding or during anticoagulant treatment, although it could be administered if the international normalized ratio (INR) is less than two [46].

## 4. Treatment of Spasticity in Spinal Cord Injury with Botulinum Toxin

### 4.1. Botulinum Toxin for Spasticity

Spasticity is one of the most common indications for BoNT. Although BoNT may be used in any type of spasticity, in general, it is considered that it should only be applied when the form of presentation is focal. The objectives that we intend with this treatment are to improve function (hand function, weight-bearing, balance, gait), ease care and positioning, prevent or reduce pain, facilitate hygiene, prevent deformities, prevent pressure ulcers, and improve the results of rehabilitation and surgery [32].

There are several clinical guidelines that recommend for the use of botulinum toxin in spasticity of brain origin, overall, in cases of CP or stroke, which are the etiologies on which most clinical trials with BoNT have been performed [51]. In fact, the official approved indications for BoNT/A1 (FDA, European drug and medical device agencies) focus on these diseases. According to the current information provided by the laboratories responsible for the commercialization and development of each presentation, ONA is indicated in the spasticity of the lower limbs when the cause is CP and in forearm and hand and leg and foot post-stroke spasticity; ABO is indicated in the spasticity of the lower limb and upper limb due to CP or stroke, and INC is indicated in upper limb spasticity.

### 4.2. Spasticity in Spinal Cord Injury

Spasticity could be treated in a standard way regardless of the etiology, but there are differences if the cause is cerebral or spinal [52]: the most frequent presentation of spasticity when the origin is spinal is generalized and diffuse, while focal spasticity is more frequent when the origin is cerebral; regarding hypertonia, patients with SCI develop more intense spasticity, the muscles most commonly affected in SCI are extensors, especially in the lower extremities, as for intrinsic phasic spasticity; extrinsic spasticity is more frequent in SCI; lower limb extensor spasms are the most prevalent spastic sign in SCI, and its most important stimulus is the hip extension (especially the last 20 degrees); finally spinal spasticity is further exacerbated by visceral diseases.

The prevalence of spasticity in SCI considering acute and chronic cases, any neurological level, and extent of injury is 65%. It can be classified according to the clinical manifestations: a tonic spasticity characterized by presenting only speed-dependent resistance to passive movement, and a phasic one characterized by the production of involuntary movements, such as clonus, hyperreflexia, and spasms [52]. The most common form of presentation is the dynamic pattern in the lower limbs (extensor spasms) that are revealed or stimulated, especially with postural shifting and transfers, and are exacerbated by triggering factors (supine position, neurogenic bladder, neurogenic bowel, pressure ulcers) [46,53]. The main patterns are shown in Table 1. These patterns show that there are too many muscles involved in SCI spasticity. As spasticity following SCI is usually generalized over the whole body, the recommended treatments are those that can correct all the affected muscles. The most important systemic treatments include oral medication (baclofen, tizanidine) and patient education (knowledge of the most frequent factors that cause a worsening of their spasticity and the times when there may be functional interference) [54,55].

If we wanted to treat the spasticity of SCI with BoNT, we would need very high doses for many several muscles, which would require an intolerable total concentration. Furthermore, there is no official indication of BoNT in spasticity of SCI. These two facts would make us think that we cannot or should not use BoNT in SCI. Some BoNT indications yet not approved by official drug agencies are contained in an international agreement, but SCI-related spasticity is not included [33]. However, a panel of experts in SCI spasticity within the International Spinal Injury Society (ISCoS) called Ability Network, have made several recommendations in the treatment of SCI spasticity, and among them is the use of BoNT/A as complementary to the rest of the treatments of which the most important is patient education and oral drugs [55]. There is only one study published as a clinical trial in SCI, comparing BoNT/A with baclofen, both treatments showing a significant decrease in tone and functional improvement [56]. However, this study failed to document the clinical characteristics of SCI (neurological level, grade of severity, functional assessment by SCI scales, etc.). The remainder of the published studies on the use of BoNT in SCI spasticity is observational studies, with a small sample size. It has been considered in the last 20 years that BoNT should be indicated for people with motor incomplete SCI (ASIA Impairment Scale grades C or D according to the International Standard Neurological Classification of SCI) because this is the condition where focal spasticity is most likely to develop, despite the fact that there was a lack of publications on the use of BoNT for SCI [57]. The largest study collected data from 90 treated patients, and it showed that BoNT/A was more effective in focal spasticity in patients with AIS D, especially if they were injected for the first time in the first 6 months of evolution, and the improvement was established in terms of a decrease in tone, pain, and joint limitations and functional improvement [58]. Systematic reviews had previously been carried out on reported cases of SCI spasticity, which were either isolated cases or mixed spastic cases due to other causes [34,59]. Marciniak et al. recorded 26 BoNT-treated SCI cases: they injected upper limb flexor muscles and antigravity muscles but found no differences in outcomes between complete and incomplete SCI or acute and chronic SCI, perhaps due to the heterogenicity of the sample [60]. Hecht et al. described 19 cases of hereditary spastic paraplegia treated with BoNT, stating that there was an improvement in tone measured with the Modified Ashworth Scale (MAS), side effects in 5 cases (weakness in 4 and pain in 1) and that the spasticity pattern was flexor (unlike the transverse SCI syndrome, which affects muscles extensors) [61]. Bermuz et al. studied 15 people with incomplete SCI and the effect of injecting 200 U of ONA into the rectus femoris measured with isokinetics muscle testing, reporting only general improvement without specifying details [62]. Other case series included spasticity of various etiologies with very small sample sizes [63,64], and the rest of the articles published include only isolated cases [65,66,67,68]. There are only a very few studies that have characterized the effect of BoNT treatment on SCI spasticity, and, therefore, well-developed clinical guidelines cannot be proposed based on strong evidence.

### 4.3. Indications for Botulinum Toxin in Spinal Cord Injury Spasticity

The indications are based on the protocols and guidelines published to date [31,32,34,46,49,55]. Focal spasticity. If the number of spastic muscles that we need to treat because they interfere with the patient’s abilities to carry out activities of daily living (ADL), walking, or transfers, is limited, we can use the BoNT.

Muscles with worse functional disadvantages. Although the spasticity may be very intense and generalized, the BoNT can be used to achieve specific partial objectives such as relaxing the muscles involved in an abnormal posture that causes a pressure ulcer or relaxing the hip adductors to allow urinary catheterizations.

- Adjuvant therapy. When spasticity is too severe and diffuse and cannot be controlled with physical therapy and various oral drugs, toxin treatment can be completed. In these cases, unlike the two previous indications, we start with the idea that we cannot achieve any objective since we cannot exceed the maximum dose of toxin that would be necessary to treat all affected muscles.

### 4.4. Assessment of Spasticity

The objective of the assessment was to measure all the types of repercussions that spasticity can have on the SCI patient to decide which is the best treatment and check whether each complication has improved or remitted after our intervention [46,69].

Tone assessment. Hypertonia can be quantified using the MAS, which is shown in Table 2. This is the main measure of spasticity and its changes after treatment, especially in spinal cord injury spasticity [70,71].

Assessment of dynamic phenomena. We can measure the frequency of spasms, but the most specific way for SCI is using the scale called Spinal Cord Assessment Tool Spastic reflexes (SCATS), which is used to measure the frequency and intensity of clonus.

Functional assessment. We must measure joint limitations and decreased power to use strength due to spasticity. It is recommended to use the SCI Independence Measure scale (SCIM III) to assess interference with ADL, and there are specific scales to assess the impact on quality of life caused by spasticity, such as Spinal Cord Injury Spasticity Evaluation Tool (SCI-SET) or Patient Reported Impact of Spasticity Measure (PRISM). If the patient has gait ability, we can quantify his problems with the Walking Index in SCI (WISCI III) and perform an instrumented gait analysis (videography, kinematics, kinetics, and electromyography).

Subjective evaluation. The patient can assess the intensity of his symptoms or interferences with a numerical scale.

Outcome measures. It is possible to evaluate the opinion that the physician or the patient have about the variations achieved in terms of each objective using the Patient/Clinician Global Impression of Change (PGIC) or to set some objectives with the patient from the beginning and assess to what extent they have been achieved after the treatment using the Goal Attainment Scaling (GAS).

### 4.5. Technical Aspects of Injection and Recommendations

The patient should sign an informed consent stating the beneficial effects we intend to achieve with the treatment, possible adverse reactions, contraindications, and interactions of the BoNT.

The location of the muscles remains mainly anatomical, although it is recommended to use some auxiliary method (electromyography, ultrasound guide), especially in the case of stroke in which flexor synergies can dislodge the muscles from their anatomical landmarks due to torsion, or when the injector has little experience [72].

The doses, in general, are recommended according to official indications and for each muscle by pharmaceutical laboratories but must be individualized according to the size of the muscle and the severity of the spasticity. In addition, it must be considered that in children below 12 years old, there are dose limits that we cannot exceed and that the first injection we make must use 50% of the estimated dose to avoid side effects due to the patient’s special predisposition. There are maximum total doses allowed for each toxin; however, due to the characteristics of the spasticity of SCI, these limits are usually exceeded [46,58]. The maximum doses and the recommended doses for the most frequent cases are shown in Table 3; they are based on previous studies and protocols, but not even the clinical trial can provide evidence for those indications and dosages [46,56,58].

After the injection, the relaxation effects can be maintained or increased with a stretching program or with splints [46,73].

If after 3 months from the previous injection, there is a better functional situation than the one before the injection, it shows that BoNT has been effective, and, therefore, we can continue with this treatment. It has been shown that over the years, the effectiveness of repeated toxin injections is not usually lost; the main studies have been done in dystonia, but it has also been evidenced in spasticity, even in SCI [46,49,74].

## 5. Conclusions

BoNT has transformed from being a powerful and dangerous poison to a safe and effective drug that can be applied in various pathologies.

Just as BoNT/A has a proven, officially indicated and protocoled use in spasticity of brain origin, it can also be used as an adjunct in the treatment of spasticity following spinal cord injuries when the manifestation of spasticity is focal. BoNT/A can also be used if the spinal cord injured patient has focal spasticity or if there are muscles with worse functional complications.

## Figures and Tables

**Table 1:** Main patterns of spasticity in spinal cord injury.

Dynamic extensor pattern of lower limbs. Although there may be hypertonia of the antigravity muscles, dynamic component predominates (extensor spasms that interfere with the transfers).
Static extensor pattern of lower limbs. It is characterized by hypertonia of the antigravity muscles and minor overactivity.
Static flexor pattern of lower limbs. A plastic muscular component predominates due to prolonged sitting, with shortening of hamstrings.
Dynamic flexor lower limbs pattern. The flexor muscles are affected, and spasms occur with the triple flexion reflex.
Upper limb flexor pattern. Muscles corresponding to flexor synergy are affected except shoulder (there is usually no internal rotation or adduction as in stroke).
Spastic paraparesis gait.

**Table 2:** Modified Ashworth Scale.

0	No increase in tone
1	Slight increase in tone with a catch or minimal resistance at the end of the range of movement (ROM)
1+	Slight increase in tone with a catch, followed by minimal resistance throughout the remainder (less than half) of the ROM
2	Marked increase in tone through most of the ROM, but the limb is easily moved
3	Considerable increase in tone; passive movement difficult
4	Limb rigid or contracted

**Table 3:** The maximum doses and the recommended doses for the most frequent cases.

MAXIMUM DOSES		ONA	ABO	INC
		400 U	1500 U	500 U
DINAMIC EXTENSOR PATTERN IN LOWER LIMBS	Adductor magnus	75 U each one	250 U each one	75 U each one
	Rectus femoris	50 U each one	150 U each one	50 U each one
	Vastus medialis	50 U each one	150 U each one	50 U each one
	Gastrocnemius (medialis)	40 U each one	100 U each one	50 U each one
STATIC EXTENSOR PATTERN IN LOWER LIMBS	Adductor magnus	75 U each one	250 U each one	75 U each one
	Rectus femoris	75 U each one	200 U each one	75 U each one
	Vastus medialis	50 U each one	150 U each one	75 U each one
	Soleus	75 U each one	200 U each one	75 U each one
STATIC FLEXOR PATTERN IN LOWER LIMBS	Adductor magnus	100 U each one	350 U each one	100 U each one
	Semitendinosus	50 U each one	150 U each one	50 U each one
	Semimembranosus	50 U each one	150 U each one	50 U each one
	Soleus	50 U each one	200 U each one	50 U each one
UPPER LIMB FLEXOR PATTERN	Biceps brachii	75 U each one	250 U each one	75 U each one
	Flexor carpi radialis	50 U each one	100 U each one	50 U each one
	Flexor profundus digitorum	50 U each one	150 U each one	50 U each one
HELPING BLADDER CATHETERIZATION	Adductor magnus	100 U each one	350 U each one	100 U each one
	Gracilis	100 U each one	150 U each one	100 U each one

ONA: Onabotulinumtoxin; ABO: Abobotulinumtoxin; INC: Incobotulinumtoxin; U: units.

## Abbreviations

ADL	Activities of daily living
SCI	Spinal cord injury
BoNT	Botulinum neurotoxin
ABO	Abobotulinumtoxin A
ONA	Onabotulinumtoxin A
INC	Incobotulinumtoxin A
CNS	Central Nervous System
CP	Cerebral palsy
MAS	Modified Ashworth Scale
